# Turning anecdotal irradiation-induced anticancer immune responses into reproducible in situ cancer vaccines via disulfiram/copper-mediated enhanced immunogenic cell death of breast cancer cells

**DOI:** 10.1038/s41419-024-06644-3

**Published:** 2024-04-27

**Authors:** Wei Guo, Lin Jia, Ling Xie, Juliann G. Kiang, Yangyang Wang, Fengfei Sun, Zunwen Lin, Enwen Wang, Yida Zhang, Peigen Huang, Ting Sun, Xiao Zhang, Zhengying Bian, Tiejun Tang, Jingtian Guo, Soldano Ferrone, Xinhui Wang

**Affiliations:** 1 Division of Gastrointestinal and Oncologic Surgery, Department of Surgery, Massachusetts General Hospital, Harvard Medical School, Boston, MA USA; 2 China Pharmaceutical University, Nanjing, China; 3 Division of Pathology, Jiangsu Province Hospital of Traditional Chinese Medicine, Nanjing, China; 4 Radiation Combined Injury Program, AFRRI USU F. Edward Hébert School of Medicine, Bethesda, MD USA; 5 Edwin L. Steele Laboratories, Department of Radiation Oncology, Massachusetts General Hospital, Harvard Medical School, Boston, MA USA; 6 Department of Orthopaedic Surgery, Massachusetts General Hospital, Harvard Medical School, Boston, USA

**Keywords:** Metastasis, Cancer microenvironment

## Abstract

Irradiation (IR) induces immunogenic cell death (ICD) in tumors, but it rarely leads to the abscopal effect (AE); even combining IR with immune checkpoint inhibitors has shown only anecdotal success in inducing AEs. In this study, we aimed to enhance the IR-induced immune response and generate reproducible AEs using the anti-alcoholism drug, disulfiram (DSF), complexed with copper (DSF/Cu) to induce tumor ICD. We measured ICD in vitro and in vivo. In mouse tumor models, DSF/Cu was injected intratumorally followed by localized tumor IR, creating an in situ cancer vaccine. We determined the anticancer response by primary tumor rejection and assessed systemic immune responses by tumor rechallenge and the occurrence of AEs relative to spontaneous lung metastasis. In addition, we analyzed immune cell subsets and quantified proinflammatory and immunosuppressive chemokines/cytokines in the tumor microenvironment (TME) and blood of the vaccinated mice. Immune cell depletion was investigated for its effects on the vaccine-induced anticancer response. The results showed that DSF/Cu and IR induced more potent ICD under hypoxia than normoxia in vitro. Low-dose intratumoral (i.t.) injection of DSF/Cu and IR(12Gy) demonstrated strong anti-primary and -rechallenged tumor effects and robust AEs in mouse models. These vaccinations also increased CD8^+^ and CD4^+^ cell numbers while decreasing Tregs and myeloid-derived suppressor cells in the 4T1 model, and increased CD8^+^, dendritic cells (DC), and decreased Treg cell numbers in the MCa-M3C model. Depleting both CD8^+^ and CD4^+^ cells abolished the vaccine’s anticancer response. Moreover, vaccinated tumor-bearing mice exhibited increased TNFα levels and reduced levels of immunosuppressive chemokines/cytokines. In conclusion, our novel approach generated an anticancer immune response that results in a lack of or low tumor incidence post-rechallenge and robust AEs, i.e., absence of or decreased spontaneous lung metastasis in tumor-bearing mice. This approach is readily translatable to clinical settings and may increase IR-induced AEs in cancer patients.

## Background

The abscopal effect (AE) denotes the ability of a localized therapy, such as irradiation (IR), to initiate a systemic antitumor response against non-irradiated metastatic cancer far outside the primary treatment area. Evidence suggests that the AE is mediated by systemic antitumor immune responses [1, 2]. Although AEs following IR have been found in many cancer types [3], they rarely occur. Even when IR was combined with immunotherapy (e.g., anti-CTLA-4 or anti-PD1 antibodies), AEs were observed in only a few patients. From 2012 to 2018 only 47 cases have been reported in patients with cancer receiving IR with or without immunotherapy [4–6]. IR induces immunogenic cell death (ICD) in various cancer cell types [7]. ICD is a phenomenon mediated by immunostimulatory signals from apoptotic cells. It is inducible by chemotherapeutics such as anthracyclines, oxaliplatin and bortezomib, as well radiotherapy, and photodynamic therapy [8–10], leading to effective antitumor immunity [11–15].

A molecular characteristic of ICD is the release or cell surface expression of highly immunostimulatory damage-associated molecular pattern molecules (DAMPs) [16], namely (i) surface exposure of calreticulin (CRT) and heat shock proteins (HSPs), (ii) passive release of high mobility group protein B1 (HMGB1), and (iii) extracellular ATP secretion. These molecules can stimulate antigen-presenting cells (APCs) as well as activating dendritic cells, leading to the development and activation of tumor-specific effector and memory T cells [16]. Disulfiram (DSF), an irreversible pan-aldehyde dehydrogenase (ALDH) inhibitor [17, 18], was approved by the FDA for the treatment of alcoholism in 1951 [19]. DSF converts to diethyldithiocarbamate (deDTC) within cells, two molecules of which bind one copper ion (Cu^2+^) to form the Cu [deDTC] complex (DSF/Cu) [20–22]. DSF/Cu targets the p97 segregase adaptor NPL4 [23], inhibits NF-κB [20, 24], and activates endoplasmic reticulum stress by upregulating the inositol requiring-enzyme 1 alpha (IRE1α)–X-box-binding protein 1 (XBP1) axis, which leads to autophagic apoptosis [25], inducing ICD in both differentiated and IR-resistant cancer stem cell (CSC) populations [26, 27].

The combination of IR and DSF/Cu has been shown to induce a more potent ICD in human breast and pancreatic cancer cells than either method alone using standard ICD parameters, including apoptosis, CRT and HSP90 cell surface expression, and the release of HMGB1 and ATP [26]. Furthermore, our recent findings have revealed that DSF/Cu and IR-stressed cancer cells/tumors possess the ability to reprogram chimeric antigen receptor (CAR) T cells, endowing them with early memory characteristics while transforming the immunosuppressive TME into an inflamed state. This reprogramming of CAR T cells and TME leads to complete rejection of tumors and establishes a durable antitumor immunological memory response in several solid tumor models [27]. Based on these findings, we tested in preclinical mouse breast cancer (BC) models whether IR and DSF/Cu-mediated enhanced ICD of BC cells can act in situ as a cancer vaccine and turn anecdotal IR-induced anticancer immune responses into reproducible AEs in cancer-bearing hosts.

## Materials and methods

### Cell lines

Mouse 4T1 and human UACC812 BC cell lines were purchased from the American Type Culture Collection (ATCC) and the human MDA-MB-231 BC cell line was acquired from the Duke Comprehensive Cancer Center Cell Culture Facility. Human MDA-MB-231 and UACC812 cells were cultured in Dulbecco’s modified Eagle’s medium (DMEM; Corning, Corning, NY, USA) supplemented with 10% heat-inactivated Gemini Foundation fetal bovine serum (FBS; Gemini Bioproducts, LLC, West Sacramento, CA, USA). The mouse HER2/neu^+^ MCa-M3C cell line, was developed at Massachusetts General Hospital, and cultured in DMEM supplemented with 15% heat-inactivated FBS [28]. The mouse triple-negative 4T1 cells [29] were cultured in RPMI 1640 medium (Corning) supplemented with 10% FBS. All the cells were cultured at 37 °C in a 5% CO_2_ atmosphere.

### Chemical reagents and antibodies

Tetraethylthiuram disulfide (disulfiram, DSF) and copper chloride (CuCl_2_ or Cu) were purchased from Sigma-Aldrich (St. Louis, MO, USA). DSF and CuCl_2_ were dissolved in DMSO and Milli-Q water, respectively. A stock solution of DSF (10 mM) was aliquoted and stored at −20 °C for up to 1 year and freshly diluted with cell culture medium (in vitro assays) or PBS (in vivo assays) prior to use. Antibodies for flow cytometry analyses were as follows: mouse anti-calreticulin antibody TO-11 and an isotype-matched mouse IgG1 MK2-23 were produced by our laboratory [30, 31], APC-Cy7-conjugated hamster anti-mouse CD3e (557596), FITC-conjugated rat anti-mouse CD4 clone H129.19 (553650), PE-Cy7-conjugated rat anti-mouse CD8a (552877), BV421 rat anti-mouse CD25 clone 3C7 (564370), PE-conjugated rat anti-mouse Foxp3 clone MF23 (560408), fixable viability stain 620 [fvs620] (564996), transcription factor buffer set (562574), anti-rat and anti-hamster Igκ/negative control compensation particles set (552845), BV650-conjugated rat anti-CD11b (563402), BV605-conjugated rat anti-CD11c (563015), and APC-conjugated rat anti-mouse Gr-1 (553129) were purchased from BD Biosciences(Franklin Lakes, NJ, USA). APC-conjugated mouse anti-human CD47 monoclonal antibody [B6H12] (17-0479-42) and APC-conjugated mouse IgG1 kappa isotype control (17-4714-82) were purchased from ThermoFisher (Waltham, MA, USA). PE-conjugated rat anti-mouse CD274 (B7-H1, PD-L1) (124308), PE-conjugated rat IgG2b, κ isotype ctrl (400635), PE-conjugated mouse anti-human CD274 (B7-H1, PD-L1) (329705), PE-conjugated mouse IgG2b, κ isotype ctrl (400311), Brilliant Violet 421™-conjugated rat anti-mouse CD273 (B7-DC, PD-L2) (107219), Brilliant Violet 421™-conjugated rat IgG2a, κ isotype ctrl (400535), Brilliant Violet 421™-conjugated mouse anti-human CD273(B7-DC, PD-L2) (329615 and Brilliant Violet 421™-conjugated mouse IgG2a, κ isotype ctrl (400259) were purchased from BioLegend (San Diego, CA, USA).

### Cell proliferation and viability assays

Tumor cells (4T1, MDA-MB-231, and UACC812) were seeded in 96-well plates at a concentration of 5 × 10^3^ cells/well in 100 μL culture medium and incubated at 37 °C and 5% CO_2_ for 24 h. Next, culture medium containing DSF/Cu at the indicated concentrations was added. Following incubation at 37 °C and 5% CO_2_ for 24 h, the proliferative/viable cells were evaluated using an MTT assay (Sigma-Aldrich). The IC_50_ values for each cell line were calculated using GraphPad Prism 8. All experiments were performed in triplicate.

### Apoptotic cell analysis

Cells (3 × 10^5^ cells/well) were seeded in 6-well plates (Corning) and treated with DSF/Cu at the indicated doses and time points. Apoptotic cells were detected by Annexin V/7-AAD Apoptosis Detection Kit (640922, BioLegend). The percentage of apoptotic cells was determined as described [26].

### Detection of CRT and immune checkpoint proteins on the cell surface

Immunofluorescence staining and flow cytometry techniques were employed to detect the translocation of calreticulin to the cell surface, as well as the expression of immune checkpoint proteins CD47, PD-L1, and PD-L2 on the cell surface, as previously described [26].

### Determination of intracellular ATP, which reflects the level of extracellular ATP

Six-well plates were seeded with 3 × 10^5^ cells/well and treated with or without DSF/Cu, as described [26].

### Detection and quantification of cytokines

HMGB1 levels were determined using a mouse HMGB1 Enzyme-linked immunosorbent assay **(**ELISA) Kit (BG-MUS11178) from Novatein Biosciences (Woburn, MA, USA) and a human HMGB1 ELISA Kit (ARG81185) from Arigobio (Hsinchu, Taiwan). TGF-β1 levels were determined using a mouse TGF beta 1 ELISA kit (ab119557) from Abcam. TNF-α, (Monocyte chemoattractant protein-1) MCP-1, IL-10 and KC (also known as (C-X-C motif) ligand 1 (CXCL1)) levels were measured with Bio-Plex Pro^TM^ Mouse Cytokine Grp I Panel 23-Plex (Mouse Cytokine 23-Plex; Bio-Rad, Hercules, California, USA). Data were analyzed using the LuminexH 100TM System (Luminex Corp., Austin, TX, USA) and quantified with MiraiBio Master-PlexH CT and QT Software (Hitachi Software Engineering America Ltd., San Francisco, CA, USA) [32].

### Irradiation

In vitro IR was performed on cells seeded in 6-well plates (3 × 10^5^ cells/well in 2 mL culture medium) at 8, 12 Gy. The X-RAD 320 Biological Irradiator (Precision X-ray, Inc., North Branford, CT) was used for all in vitro and in vivo IR experiments.

### In situ tumor vaccination

Six-week-old female BALB/c and FVB mice were purchased from the Massachusetts General Hospital COX7 animal facility. To establish tumors, a single-cell suspension of mouse 4T1 cells (3.5 × 10^5^ per mouse) in RPMI1640 serum-free medium was subcutaneously (s.c.) injected into the hind legs of mice (day 0). When the tumors were palpable, we initiated a cycle of in situ tumor vaccination consisting of 3-site intratumoral injections of DSF/Cu (at the indicated doses in 100 μL PBS on days 4 and 6) and a single dose of IR (12 Gy) delivered locally to each mouse tumor (on day 5). The same vaccination cycle was repeated on day 9. On day 9, the mice were rechallenged with 5 × 10^5^ 4T1 cells injected s.c. into the other side of the hind leg. The FVB mice were inoculated orthotopically with 1 × 10^6^ MCa-M3C cells/per mouse in the left mammary fat pad and treated with DSF/Cu (1.5 μM/1 μM) and IR (12 Gy for cycles 1, 2, and 8 Gy for cycle 3). We measured tumor growth daily with a caliper and calculated the volume according to the following formula: V = 1/2 (longer diameter long × shorter diameter^2^). At time of sacrifice, the tumors, lungs, and spleens were collected for analysis of immune cell subtypes, cytokines and cellular level metastasis. All animal studies were approved by the Institutional Animal Care and Use Committee.

### Mouse primary tumor and lung metastasis sample preparations for histologic and flow analyses

For hematoxylin and eosin (H&E) staining, entire lung tissues (4T1 mouse model) were collected from each mouse at sacrifice and formalin-fixed and paraffin-embedded (FFPE). For flow cytometry analysis or detection of lung metastatic cancer cells using cell culture techniques, each tumor or lung (50% tissue of each lung, MCa-M3C mouse model), and spleen was collected at sacrifice. Primary tumors or lungs were minced into 3 × 3 mm pieces and digested with Collagenase IV (LS004188) (1 mg/mL PBS) (Worthington Biochemical Corp, Lakewood, NJ, USA.) at 37 °C for 1 h. Spleens were mechanically homogenized using a fine metal mesh net and then the red blood cells in the splenocyte suspension were lysed with ammonium-chloride-potassium (ACK) lysing. The digested tumor or splenocyte suspension was filtered through a 40 μM cell strainer to obtain single-cell suspensions for flow analysis.

### Statistical analysis

Unless otherwise noted, data are presented as the mean ± SEM. We used one-way ANOVA, two-way ANOVA, studentized range test, and Student’s *t*-test to compare groups and paired samples. All statistical analyses were performed using GraphPad Prism 8. Differences were considered statistically significant when the *p*-value was <0.05.

## Results

### DSF/Cu induced ICD of mouse and human BC cells in a dose- and time-dependent manner

**Fig. 1:**
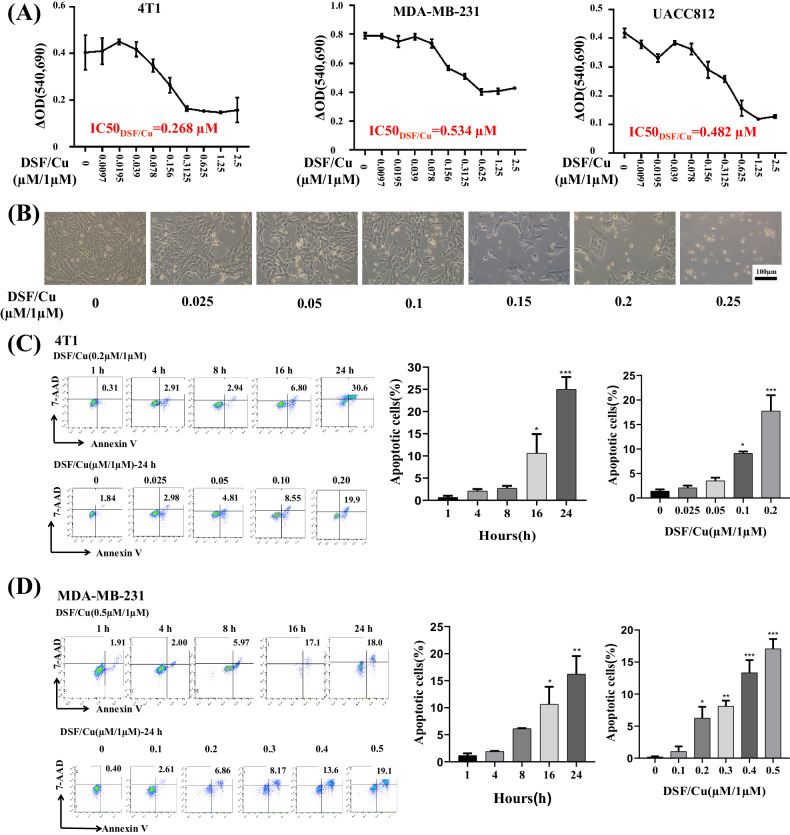
DSF/Cu was cytotoxic to mouse and human BC cells in vitro. **A** Mouse 4T1, human MDA-MB-231, and UACC812 BC cells were treated with the indicated concentrations of DSF and CuCl_2_ (1 μM). After 24 h of exposure, cell viability was determined via MTT assays. The DSF/Cu IC50 of each cell line is indicated. **B** Death of 4T1 cells treated with DSF/Cu observed via visual microscopy. **C**, **D** 4T1 (**C**) and MDA-MB-231 (**D**) BC cell lines were treated with DSF/Cu for the indicated times, and the late-stage apoptotic (Annexin V^+^/7-AAD^+^) cells were determined using flow cytometry. Data of late apoptosis are presented as the mean ± SEM of three replicates from one representative experiment. ***p < 0.001, **p < 0.01 and *p < 0.05 vs control group. The experiments were performed 3 times.

**Fig. 2:**
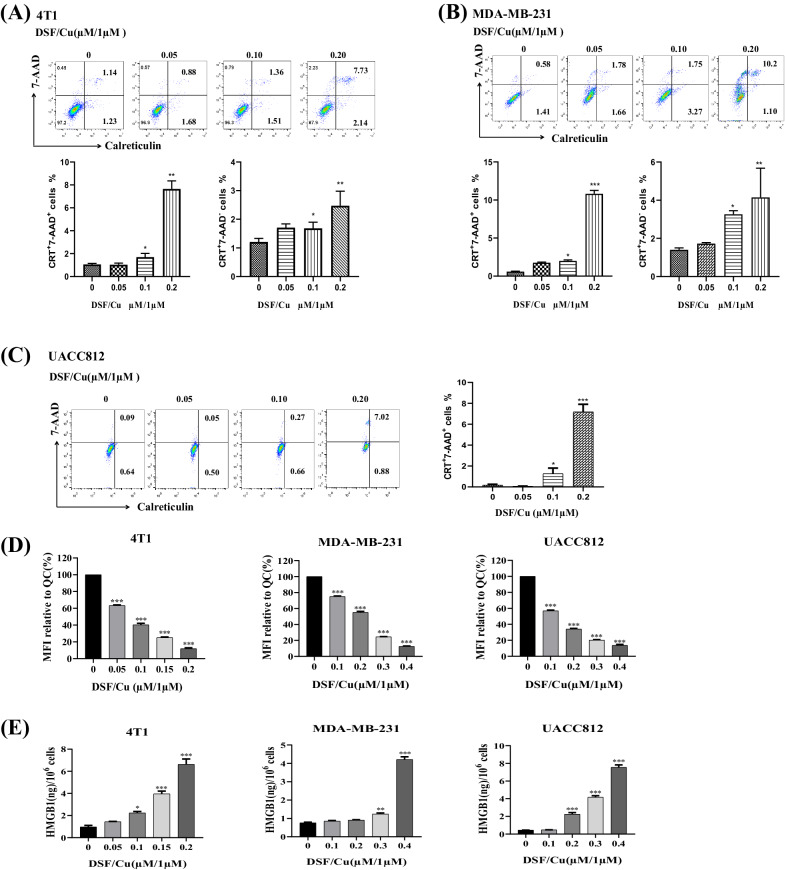
DSF/Cu-induced ICD in vitro. **A**–**G** Cells were treated with DSF at the indicated concentrations and CuCl_2_ (1 μM) for 24 h in vitro. **A**–**C** Mouse 4T1 (**A**), human MDA-MB-231 (**B**), and UACC812 (**C**) BC cells were harvested, and aliquots were subjected to flow cytometry analysis for ICD, measured as calreticulin (CRT)^+^ and 7-AAD^+^ cells. **D** Decreased intracellular ATP, reflecting increased extracellular ATP, measured by staining cells with quinacrine (QC). MFI of the FL1 channel (for QC) was determined by flow cytometry and the relative intracellular ATP content was expressed as the percentage of MFI relative to untreated cells. **E** Cell culture supernatants from cells treated as indicated were analyzed using enzyme-linked immunoassay (ELISA) for extracellular HMGB1 protein levels. Data are presented as the mean ± SEM of three replicates from one representative experiment. ***p < 0.001, **p < 0.01, and *p < 0.05, individual DSF/Cu-treated groups vs. untreated group. The experiments were performed 3 times.

Exposure to DSF (0.01–2.5 μM) and a fixed concentration of CuCl_2_ (1 μM) for 24 h in vitro of mouse 4T1 and human MDA-MB-231 and UACC812 BC cells indicated that the half-maximal inhibitory concentration (IC_50_) value of DSF was 0.268 μM for 4T1, 0.534 μM for MDA-MB-231, and 0.482 μM for UACC812 cells (Fig. 1A). When 4T1 cells were observed by optical microscopy, the tumor cells started to appear round when treated with 0.025–0.25 μM DSF and CuCl_2_ (1 μM) and became prominent at concentrations ≥0.15 μM, with more floating cells (Fig. 1B). Flow cytometry analysis revealed that the increase in apoptotic cells was DSF/Cu dose-dependent and time-dependent (Fig. 1C, D). The molecular characteristics of ICD were assessed in DSF/Cu-treated cells by determining their cell-surface expression or release of highly immunostimulatory DAMPs, including, CRT, released ATP levels, and extracellular HMGB1. DSF/Cu induced CRT cell surface expression in a dose-dependent manner more prominently in dying or dead (7-AAD^+^) than viable (7-AAD^-^) 4T1 and MBA-MD-231 cells (Fig. 2A–C). DSF/Cu decreased intracellular ATP levels, reflecting increased extracellular release of ATP [26] in a dose-dependent manner for all three cell lines (Fig. 2D). DSF/Cu increased the release of extracellular HMGB1 in a dose-dependent manner in all three cell lines (Fig. 2E). Furthermore, we assessed the cell surface expression levels of inhibitory immune checkpoint proteins, including CD47, PD-L1, and PD-L2 [33, 34], on cells treated with DSF/Cu. Baseline expression of CD47 and PD-L1 was naturally high in untreated MDA-MB-231 and UCAA812 cells. Treatment with DSF/Cu led to a dose-dependent reduction in CD47 and PD-L1 expression on viable cells (7-AAD-) (Supplementary Figs. 1A, B, E, F and 2A, B, E, F). In contrast to untreated controls, DSF/Cu treatment caused a decrease in CD47 and PD-L1 levels on dying or dead cells (7-AAD^+^) in both cell lines (Supplementary Figs. 1C, G and 2C, G).Notably, the decrease in CD47 and PD-L1 expression on dying or dead cells (7-AAD^+^) showed an inverse correlation with DSF/Cu dosage, indicating that higher doses resulted in a smaller reduction of these expressions (Supplementary Figs. 1D, H and 2D, H). This unexpected trend suggests that the relationship between DSF/Cu dosage and the modulation of immune checkpoint expressions is complex and warrants further investigation for a comprehensive understanding. Nonetheless, the net reduction of CD47 and PD-L1 on dying/dead and viable tumor cells induced by DSF/Cu may contribute to an enhanced ICD. There was no detectable PD-L2 cell surface expression before or after DSF/Cu treatment (Supplementary Fig. 3).

### DSF/Cu and IR induced more potent ICD in BC cells under hypoxia than under normoxia

**Fig. 3:**
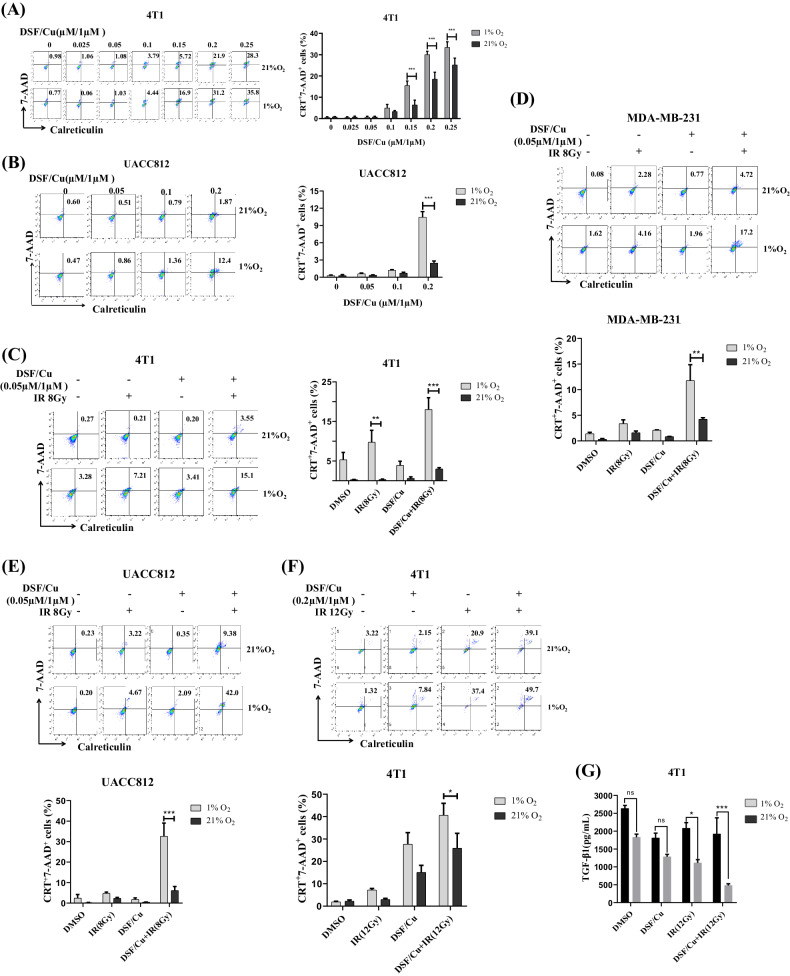
DSF/Cu and IR induced more potent ICD of BC cells under hypoxia than under normoxia in vitro. Mouse 4T1, human MDA-MB-231, and UACC812 BC cells were treated with DSF/Cu and IR or DSF/Cu or IR alone under normoxic (21% O_2_) or hypoxic (1% O_2_) conditions. DSF was used at the indicated concentrations with CuCl_2_ (1 μM). **A**, **B** 4T1 (**A**) and UACC812 (**B**) cell lines were treated with various DSF/Cu concentrations for 24 h under normoxic or hypoxic conditions. They were then harvested, and aliquots were analyzed for 7-AAD^+^ and cell surface calreticulin^+^ cells using flow cytometry. **C**, **D**, **E** 4T1 (**C**) MDA-MB-231 (**D**) and UACC812 (**E**) cells were treated with a very low dose of DSF/Cu (0.05 μM/1 μM) and 8 Gy IR under normoxic or hypoxic conditions and analyzed for 7-AAD^+^ and cell surface calreticulin^+^ cells using flow cytometry. **F**, **G** After treating 4T1 cells with DSF/Cu (0.2/1 μM) for 1 h, they were subjected to 12 Gy IR. Subsequently, the cells were exposed to an additional 1-hour treatment of DSF/Cu before being incubated under normoxic or hypoxic conditions for 24 h and analyzed for 7-AAD^+^ and cell surface calreticulin^+^ cells and their culture supernatants by ELISA for transforming growth factor (TGF)-β1 expression. For all calreticulin flow staining experiments, an isotype matched control antibody was used as a specificity control. Data are presented as the mean ± SEM of three replicates from one representative experiment. ns indicates no significant difference; ***p < 0.001, **p < 0.01 and *p < 0.05 vs. the indicated group. The experiments were performed 3 times.

Hypoxia is a hallmark of all solid tumor microenvironments (TME) and is strongly associated with tumor resistance to chemotherapy and IR [35, 36]. Thus, it was vital to determine whether DSF/Cu can induce ICD under hypoxia. Initially, we investigated whether DSF/Cu could induce ICD, determined as 7-AAD^+^ and cell surface CRT^+^ cells, in BC cells under a hypoxic (1% O_2_) condition. When 4T1 and UCAA812 cells were tested, DSF/Cu-induced ICD was detected as 7-AAD^+^ CRT^+^ cells in a dose-dependent manner under hypoxic and normoxic (21% O_2_) conditions (Fig. 3A, B). The extent of DSF/Cu-induced ICD was more pronounced under hypoxia than normoxia in both cell lines (Fig. 3A, B). These data and our previous finding that DSF/Cu made IR-resistant BC stem cells (BCSCs) as sensitive to IR-induced ICD as non-BCSCs under normoxia prompted a new investigation, namely whether the combination of DSF/Cu and IR under hypoxia could also synergistically induce potent ICD in BC cells [26]. Indeed, DSF/Cu (a low-dose of 0.05 μM/1 μM) and a single dose (8 Gy) IR induced more ICD under hypoxia than under normoxia in all three cell lines (Fig. 3C–E). Next, we tested whether similar results could be obtained using different doses of DSF/Cu (0.2 μM/1 μM) and IR (12 Gy). As expected, under hypoxia, such doses of DSF/Cu and IR tended to induce more potent ICD of 4T1 cells than under normoxia (Fig. 3F). 4T1 cells treated under hypoxic conditions produced less TGF-β1, a critical cytokine involved in the regulation and suppression of immune responses (Fig. 3G). These results provide a firm rationale to test the hypothesis that a combination of DSF/Cu and IR may convert tumors into an in situ vaccine through the induction of strong ICD of tumor cells and reverse immunosuppressive TME in vivo.

### Combination of i.t. injection of DSF/Cu and localized tumor IR elicited a robust antitumor immune response in immunocompetent mice

**Fig. 4:**
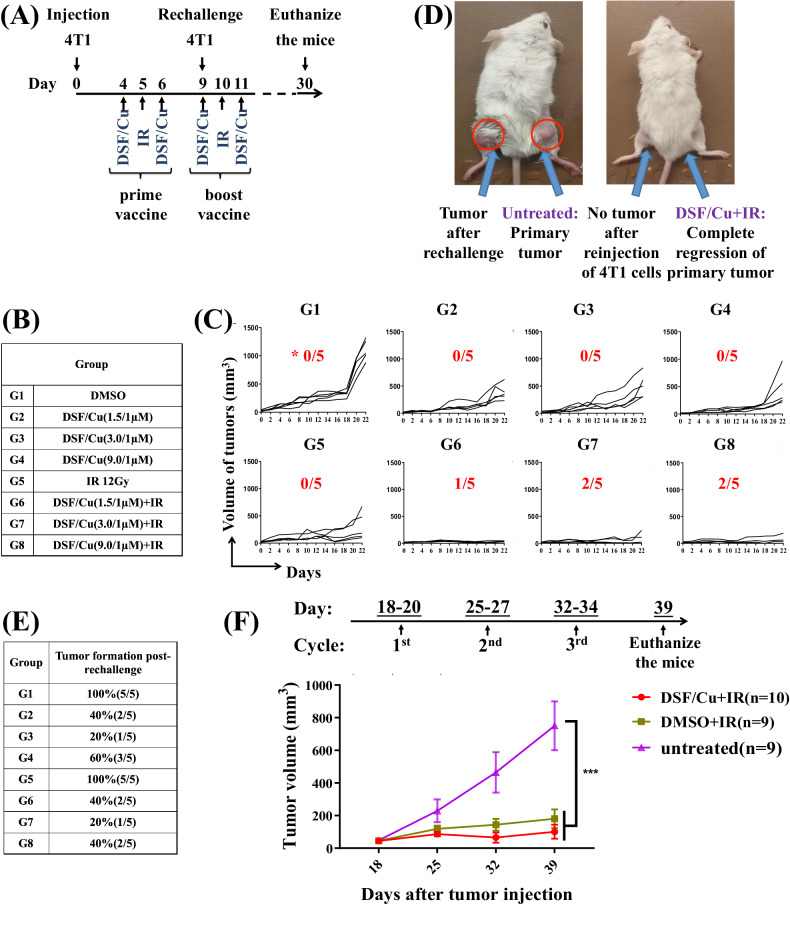
DSF/Cu combined with IR induced potent immune response and immunological memory responses against primary and rechallenged tumor. **A** Scheme of the experimental design for the eight groups of 4T1 tumor-bearing mice treated with in situ cancer vaccination. **B** Groups of 4T1 tumor-bearing mice with different treatments. **C** Tumor growth or rejection in the 4T1 model was assessed on the indicated days in the eight groups of each mouse. *The number of complete tumor rejections in each group (*n* = 5) is shown. **D** A photograph (left) represents 4T1 primary tumor growth in the tumor in the right leg and the same tumor cell-rechallenged tumor. The photograph (right) shows primary tumor rejection after in situ cancer vaccination (DSF/Cu (1.5/1 μM) i.t. + IR) and tumor rejection after reinjection of 4T1 cells into the untreated right leg of the same mouse. Photos were taken 30 days after the initiation of primary 4T1 cell inoculation. **E** The percentages of tumor incidence in the groups of mice after 4T1 tumor cell rechallenge (5 × 10^5^ cells/mouse). **F** MCa-M3C (1 × 10^6^/ mouse, inoculated orthotopically) tumor-bearing mice were treated with in situ cancer vaccination (i.t DSF/Cu at 1.5/1 μM + IR) or IR only or left untreated. The average volumes of tumors expressed as mean ± SEM in each group are shown. ***p < 0.001 vs. the indicated group.

Based on the data shown in Fig. 3, we reasoned that localized delivery of both DSF/Cu and IR could induce ICD effectively within the tumor, and that cells that undergo strong ICD should elicit a systemic antitumor immune response, resulting in regression of primary tumor growth and prevention of metastasis. To this end, after an initial dose titration in vivo, the efficacy of low-doses of DSF (1.5, 3, and 9 μM), with a fixed Cu dose (1 μM) was tested by multiple site i.t. injections of 100 μL PBS containing DSF/Cur, given one day before and after localized tumor IR(12Gy). This regimen, designed as an in situ cancer vaccine, was administered to syngeneic 4T1-derived tumors growing subcutaneously s.c. in the right hind legs of mice (Fig. 4A, B). In total, 33.3% (5/15) of the mice receiving the in situ cancer vaccine of DSF/Cu+IR (all 3 DSF/Cu doses) exhibited complete tumor rejection (Fig. 4C, D), whereas none of the 4T1-bearing mice receiving either vehicle, DSF/Cu or IR alone had complete tumor rejection (Fig. 4C, D). As expected, this approach resulted in an immunological memory response in the same set of mice, indicated by significantly reduced tumor formation (20–40%) than that in mice treated with IR only (100%) after 4T1 cell rechallenge (Fig. 4E). We repeated the same in situ vaccination approach for syngeneic MCa-M3C-derived tumors growing orthotopically in the left mammary fat pad of FVB mice. Tumor volumes remained steadily smaller in mice treated with DSF/Cu (1.5 μM/1 μM) and IR (12 Gy) or IR alone than in those treated with DMSO (Fig. 4F). The difference between these two groups was smaller than that in the 4T1 tumor model, perhaps because MCa-M3C is more sensitive to IR-mediated killing than 4T1, which is known to be radioresistant [29, 37]. Overall, combined treatment of DSF/Cu and IR had a therapeutic effect on multiple types of BC in mice.

### In situ cancer vaccine of DSF/Cu and IR resulted in profound AEs on lung metastasis

**Fig. 5:**
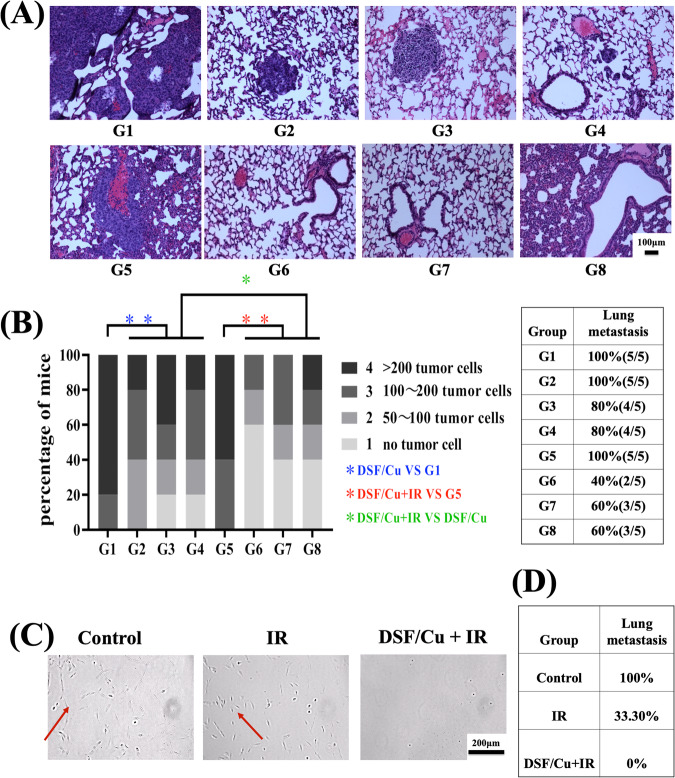
DSF/Cu combined with IR induced robust AEs. Scheme of the experimental design is indicated in Fig. 4A, B. At the time of euthanasia, mouse lungs were collected and assessed for metastasis. **A** Representative spontaneous lung metastatic burden shown on H&E-stained formalin-fixed and paraffin-embedded (FFPE) lung tissues harvested on day 30. **B** Quantitative lung metastatic burden, defined by the number of metastasized tumor cells, of mice in each experimental group with grades 1–4 scored as indicated on H&E-stained FFPE sections. The bar, at a scale of 1–4, is the highest tumor grade present in each mouse. **C** Detection of metastatic tumor cells in the lungs by culturing lung cell suspensions collected on day 39 from MCa-M3C tumor-bearing mice treated as indicated above (Fig. 4F). **D** Percentages of lung metastasis in the groups of mice bearing MCa-M3C tumors treated as indicated. **p < 0.01 and *p < 0.05 vs. control group or between indicated groups.

The antitumor immune response against primary tumors observed in highly metastatic 4T1 and MCa-M3C mouse tumor models led us to investigate whether such an immune response would have AEs, that is, whether tumor growth would be reduced outside the local treatment fields of DSF/Cu and IR [2]. The lungs are common metastatic target sites for BC in humans. We employed the 4T1 and MCa-M3C mouse tumor models for our study. These models are known for their high rate of spontaneously developing lung metastases following the primary implantation of tumor cells into the orthotopic mammary fat pad or subcutaneously [28, 29, 38,]. Therefore, at the time of euthanizing tumor-bearing mice and treated as described in Fig. 4A, B, the lungs were collected. A formalin-fixed and paraffin-embedded lung tissue was consecutively sectioned from each mouse. Then, these sections were stained with hematoxylin and eosin (H&E), followed by microscopic examination, allowing for the definitive identification of cancerous cells. This histopathological examination can quantitatively confirm the presence or absence of metastasis, which is characterized by the complete absence of cancer cells (zero cancer cells). This condition was predominantly observed in mice treated with DSF/Cu+IR, and to a lesser extent in those receiving DSF/Cu treatment (Fig. 5A, B). The overall metastatic incidence of all DSF/Cu+IR treated groups (40–60%) was lower than that of either the IR (100%) or DSF/Cu alone treated group (80–100%) (Fig. 5B). In addition, primary tumors treated with DSF/Cu+IR showed extensive AEs by complete prevention of lung metastasis in 100% of MCa-M3C tumor-bearing mice, as detected through a sensitive lung tissue culture technique (Fig. 5C, D). The AEs may be attributed to the degree of elimination of BCSCs and ICD induced by DSF/Cu in primary tumors [26] followed by systemic immune responses as elucidated below.

### Antitumor efficacy of the in situ cancer vaccine of DSF/Cu and IR was immune effector CD8^+^ and CD4^+^ T cell-dependent and modulated by suppressive cytokines in the TME and peripheral blood of mice

**Fig. 6:**
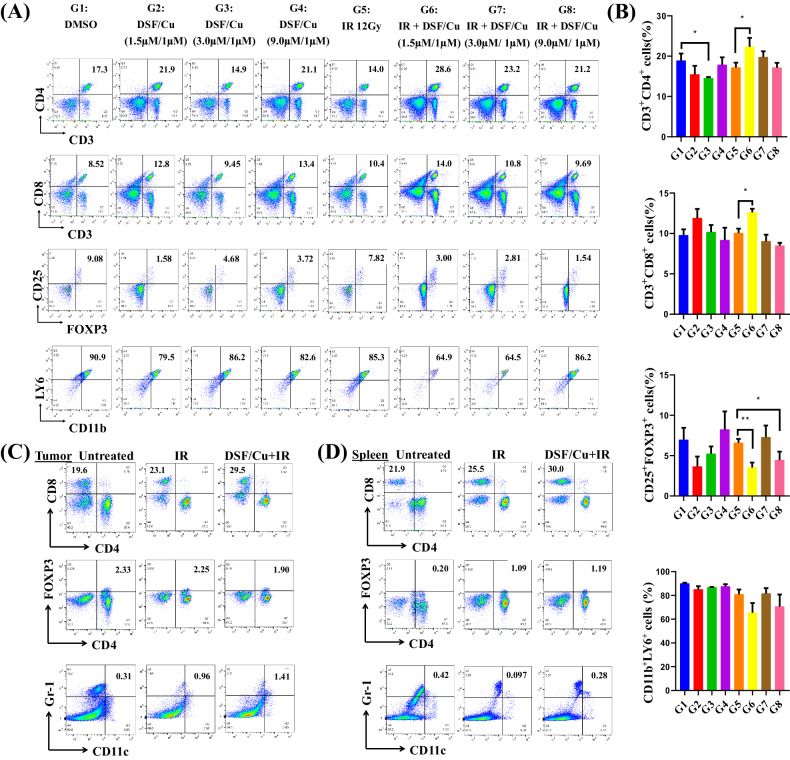
DSF/Cu and IR induced robust immune responses in 4T1 and MCa-M3C tumor-bearing mice. Scheme of the experimental design is indicated in Fig. 4A, B. **A**, **B** Flow cytometry analysis of splenocytes obtained on day 30 from each group of 4T1 tumor-bearing mice for fvs620^-^CD3^+^CD4^+^ (CD4^+^T) cells, fvs620^-^CD3^+^CD8^+^ (CD8^+^T) cells, fvs620^-^CD3^+^CD4^+^ CD25^+^FOXP3^+^ (Treg) cells, and fvs620^-^ CD11b^+^LY6^+^ (MDSCs). Representative flow analysis of immune subsets detected in the splenocytes of one mouse of each experimental group is shown. Percentages of CD4^+^ T cells, CD8^+^ T cells, Tregs, and MDSCs present in splenocytes from the eight experimental groups of mice (**B**). **C**, **D** Flow cytometry analysis for CD4^+^ T cells, CD8^+^ T cells, Tregs, and dendritic cells (DCs) in the tumor (**C**) and spleen (**D**) from 2–3 pooled samples obtained from each group of MCa-M3C tumor-bearing mice. Data are represented as the mean ± SEM; *n* = 3–5 from a representative experiment. ns indicates no significant difference; **p < 0.01, and *p < 0.05 vs. indicated group.

To analyze the ICD-elicited immune response of cancer cells, immune cell subtypes in the spleen or TME (if tumor tissues were available) were assessed. In the 4T1 model (Fig. 4A, B), we observed in splenocytes obtained from DSF/Cu+IR-treated mice compared to those of mice treated with IR alone increased numbers of CD8^+^ and CD4^+^ T cells and decreased Treg cell numbers (CD25^+^FOXP3^+^), and potentially decreased myeloid-derived suppressor cells (^+^CD11b^+^ Ly6^+^) (Fig. 6A, B). In the MCa-M3C mouse model, increased CD8^+^ T cell and DC (Gr-1^+^CD11c^+^) numbers and decreased Treg cell numbers (CD4^+^FOXP3^+^) in tumor tissues, and increased CD8^+^ T cell numbers in spleens were found in IR + DSF/Cu-treated mice (Fig. 6C, D).

**Fig. 7:**
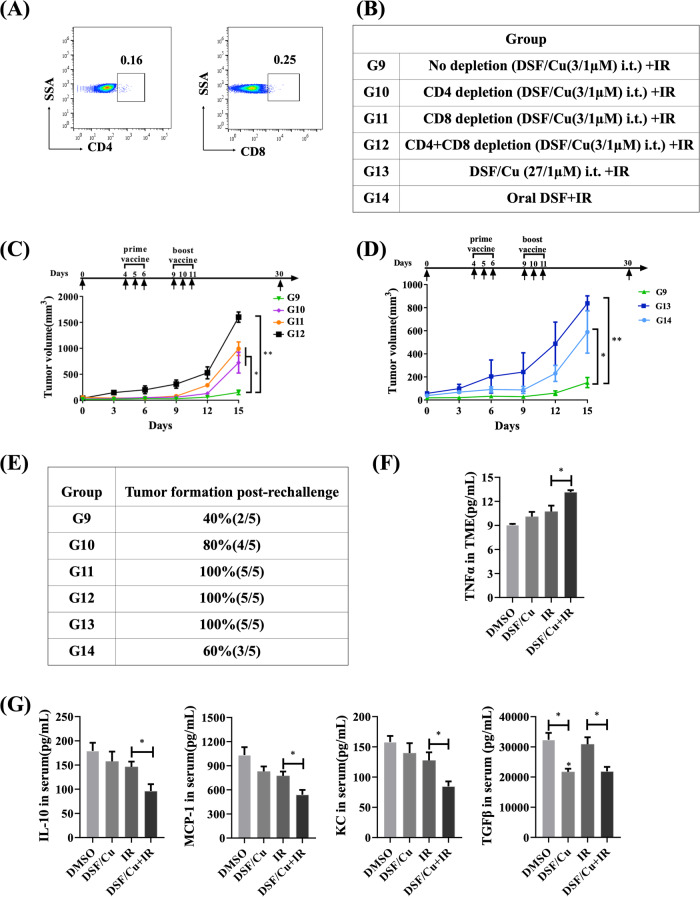
Antitumor efficacy of the in situ cancer vaccine by localized delivery of DSF/Cu and IR was determined by immune effector CD8^+^ and CD4^+^ T cell-dependent and modulated chemokines/cytokines in the TME and peripheral blood of mice. Scheme of the experimental design is indicated in Fig. 4A, B. **A**–**D**: **A** Results of immunodepletion of CD4^+^ and CD8^+^ cells from peripheral blood in 4T1-bearing mice. **B** Groups of 4T1 tumor-bearing mice with different treatments. **C** 4T1 tumor-bearing mice 1-day prior in situ cancer vaccination (DSF/Cu at 3/1 μM + IR) were divided randomly into four groups as follows: no depletion, CD4^+^ cell depletion, CD8^+^ cell depletion, and both cell depletion by anti-mouse CD4 or CD8 monoclonal antibodies (from Biocell.com). **C** The average volumes of tumors expressed as mean ± SEM in each group are shown. **D** Comparisons of antitumor efficacy in 4T1-bearing mice treated in situ cancer vaccination (DSF/Cu at 3 μM /1 μM + IR) vs. a high dose DSF/Cu (27 /1 μM + IR) vs. orally administered DSF (4 mg/kg) daily for 7 days and IR. The average volumes of tumors expressed as mean ± SEM in each group are shown. **E** Percentage of tumor incidence in each group after 4T1 tumor cell rechallenge. **p < 0.01 and *p < 0.05 vs. control group or between indicated groups. **F**, **G** The levels of panels of mouse cytokines/chemokines were measured by MSD multiplex immunoassays. **F** Immune-promoting cytokine TNFα was present in the TME and **G** Serum immunosuppressive chemokines/cytokines were detected (IL-10, MCP-1, KC, and TGFβ) from each group of 4T1 tumor-bearing mice treated as indicated. Data are presented as the mean ± SEM; *n* = 5 from a representative experiment. *p < 0.05 compared with the indicated groups.

Next, whether the antitumor efficacy of the in situ cancer vaccine was mediated by immune effector cells (Fig. 7A) in the 4T1 mouse model via depleting CD8^+^ and/or CD4^+^ T cells was evaluated. Depletion of both CD8^+^ and CD4^+^ T cells completely abolished the anti-primary and -rechallenged tumor responses elicited by the in situ cancer vaccine, while depletion of either cell type caused only a modest reduction (Fig. 7B, C). Previously, we performed an initial DSF/Cu dose titration in vitro experiment and found that DSF/Cu is toxic to peripheral blood mononuclear cells (PBMC) at a dose of ~0.15 μM/1 μM, which decreased ~50% PBMC in vitro. To confirm only a low-dose of DSF/Cu can be used to avoid toxicity to immune cells continuously recruited from blood to TME, we tested DSF/Cu at 27 μM/1 μM, which is 3 times higher than 9 μM/1 μM, i.t. administered instead of lower DSF doses (1.5–9 μM/1 μM) in the 4T1 tumor model. Such a high DSF/Cu dose, which is highly likely toxic to immune cells in TME, combined with IR diminished all anti-primary and—rechallenged tumor effects in the treated mice (Fig. 7D, E). However, the exact optimal dose range of i.t. delivery DSF/Cu has yet to be determined.

Moreover, DSF/Cu i.t. delivery was more effective than oral low-dose DSF oral delivery, which only produced modest anti-primary and—rechallenged tumor effects (Fig. 7D, E). However, more dose ranges of DSF/Cu with administered routes as well as IR regimen should be further tested for optimization. Lastly, the chemokines/cytokines in serum and tumor collected from each mouse treated, as indicated in Fig. 4B, analyzed were IL-1α, IL-1β, IL-2, IL-3, IL-4, IL-5, IL-6, IL-9, IL-10, IL-12(p40), IL-12(p70), IL-13, IL-17, eotaxin, G-CSF, GM-CSF, IFN-γ, KC, MCP-1, MIP-1α, MIP-1β, RANTES and TNF-α (Supplementary Table 1). The in situ cancer vaccination with DSF/Cu+IR increased proinflammatory cytokine TNF α level in TME and downregulated expression serum levels of immunosuppressive chemokines/cytokines including IL-10, MCP-1, KC, and TGF-β in peripheral blood in 4T1 tumor-bearing mice (Fig. 7F, G), indicating its potential to reverse the immunosuppressive TME (cold tumor) into inflamed TME (hot tumor).

## Discussion

Prevention and treatment of metastatic BC are urgent and unmet clinical needs since it is a major contributor to cancer-related mortality, even in patients diagnosed with early-stage disease. Most BC patients are diagnosed at early stages (Stages I, IIA, IIB, and IIIA) [39, 40]. However, while current therapies achieve significant local control of the disease, ~30% of patients with early-stage disease eventually develop metastatic BC [41].

Radiation therapy (RT) uses intense energy beams to kill cancer cells by damaging their DNA. Approximately 50% of patients with cancer receive RT [42]. RT is the mainstay of treatment for BC at almost every stage, as it is an effective way to reduce the risk of post-surgery recurrence for stage I–III cancers and alleviate the symptoms caused by stage IV metastatic BC [43]. Recently, radiation therapy has evolved from a local to a systemic therapy for cancer, owing to its ability to regulate the immune response. Potentially RT or IR can augment immune responses against both the target tumor and metastatic sites by modulating antitumor immunity through the release of tumor antigens, tumor DNA, and cytokines into the TME and induction of ICD [26, 44]. However, RT alone rarely induces strong systemic antitumor immune responses, such as AEs [4]. Indeed, when IR is combined with immunotherapy using the anti-CTLA4 monoclonal antibody, ipilimumab, AEs have occurred in melanoma and metastatic non-small cell lung cancer [1, 45]. Nevertheless, these are rare anecdotal clinical events [46]. To meet this unmet clinical challenge, combined IR and immunotherapy has gained considerable interest from researchers. Several studies have used IR for in situ tumor vaccination, in which a patient’s tumor is used as a source of tumor antigens to stimulate effective antitumor immune responses. The advantage of this approach is to generate antitumor immune responses using the most immunogenic, T cell-recognizable, and diversified tumor antigens, which are termed “private antigens.” Private antigens are derived from patient-specific mutated and differentiated proteins in a tumor. The immunity elicited by in situ tumor vaccination is expected to target all or multiple tumor cancer antigens; thus, this approach may address the challenge of treating heterogeneous cancers [47, 48]. Morris et al. reported that combining IR and an intratumoral IL2-linked tumor-associated antigen-specific antibody (anti-GD2 Hu14.18K322A or anti-EGFR cetuximab) in mouse models of melanoma, neuroblastoma, and head and neck squamous cell carcinoma eradicated both large tumors and metastases and elicited T cell immune responses against primary tumors that can be further leveraged by anti-CTLA-4 T cell checkpoint blockade to reduce lung metastasis [48, 49]. Demaria et al. identified DNA exonuclease Trex1 as being an upstream regulator of IR-induced antitumor immunity. A proper dose of radiation-induced Trex1 induction, which activates the type-I interferon (IFN-I) pathway mediated via cyclic GMP-AMP (cGAMP) synthase (cGAS) and its downstream adaptor stimulator of interferon genes (STING), can optimally stimulate antitumor-specific CD8^+^ T responses. When IR (8 Gy × 3) was combined with the immune checkpoint inhibitor (ICI) anti-CTLA-4, complete and durable regression of both the irradiated and non-irradiated mouse cell line TSA-derived tumor was observed [15]. Greenberg et al. also demonstrated abscopal responses driven by anti-CTLA4 therapy and vaccination of IR-treated mouse B16 melanoma cells via the pattern-recognition receptor cGAS-STING axis [50].

Notably, all these studies involved ICIs or immune checkpoint blockers (ICBs), which have been revolutionary drugs for many cancers. However, ICIs have serious limitations: (i) only a minority patients receive long-term benefits (i.e., objective responders), (ii) their use is limited by their toxicity, because severe adverse immune-related events occur, which can be irreversible and sometimes life-threatening, and (iii) they may be too expensive for many patients. Our study report that the in situ vaccine composed of IR with DFS/Cu in the absence of ICIs offers the following unique characteristics: (i) it enhances IR-induced non-antigen/target-dependent ICD in many cancer types, including BC, pancreatic cancer, osteosarcoma, sarcoma, and melanoma [26] (Data not shown); (ii) the in situ vaccine alone induces profound reproducible AEs, measured by clinical resembling spontaneous lung metastasis in two mouse BC models; (iii) our earlier data demonstrated that it could induce ICD in differentiated or differentiating breast non-cancer stem cells and BCSCs, which are the root cause of cancer formation, progression, and metastasis [26, 27] and robust immune responses against such a broad spectrum of tumor antigens, including antigen sources derived from BCSCs, may also explain why this approach could achieve robust reproducible AEs.

This research showed that low-dose i.t. delivery of DSF/Cu, combined with tumor-localized IR, act as an effective in situ cancer vaccine. This approach triggered significant antitumor immune responses, both against primary tumors and upon tumor rechallenge, and induced robust AEs in mouse models. Presently, we are developing tumor targeting antibody-expressing nanovesicles for systemic delivery of DSF/Cu directly to tumors. This strategy is particularly aimed at cases where tumors are not accessible for i.t. injection of DSF/Cu.

Our findings hold great promise for rapid translation of this simple approach that can use a single fraction of IR (12 Gy) with i.t. delivery of a low-dose of the FDA-approved DSF (e.g., 1.5 μM) with copper (1 μM) to turn anecdotal IR-induced anticancer immune responses into reproducible AEs for BC patients.

### Supplementary information

Supplementary Figure 1

Supplementary Figure 2

Supplementary Figure 3

Supplementary Table 1

Legends for supplementary figures and table

## Data Availability

The original data and cell lines that supporting the conclusion of this article are available upon request.
