# Supplementary figures and images for: Turning anecdotal irradiation-induced anticancer immune responses into reproducible in situ cancer vaccines via disulfiram/copper-mediated enhanced immunogenic cell death of breast cancer cells
Source: Cell Death Dis. 2024 Apr 27;15(4):298. doi: 10.1038/s41419-024-06644-3 (PMC11055882; doi:10.1038/s41419-024-06644-3)

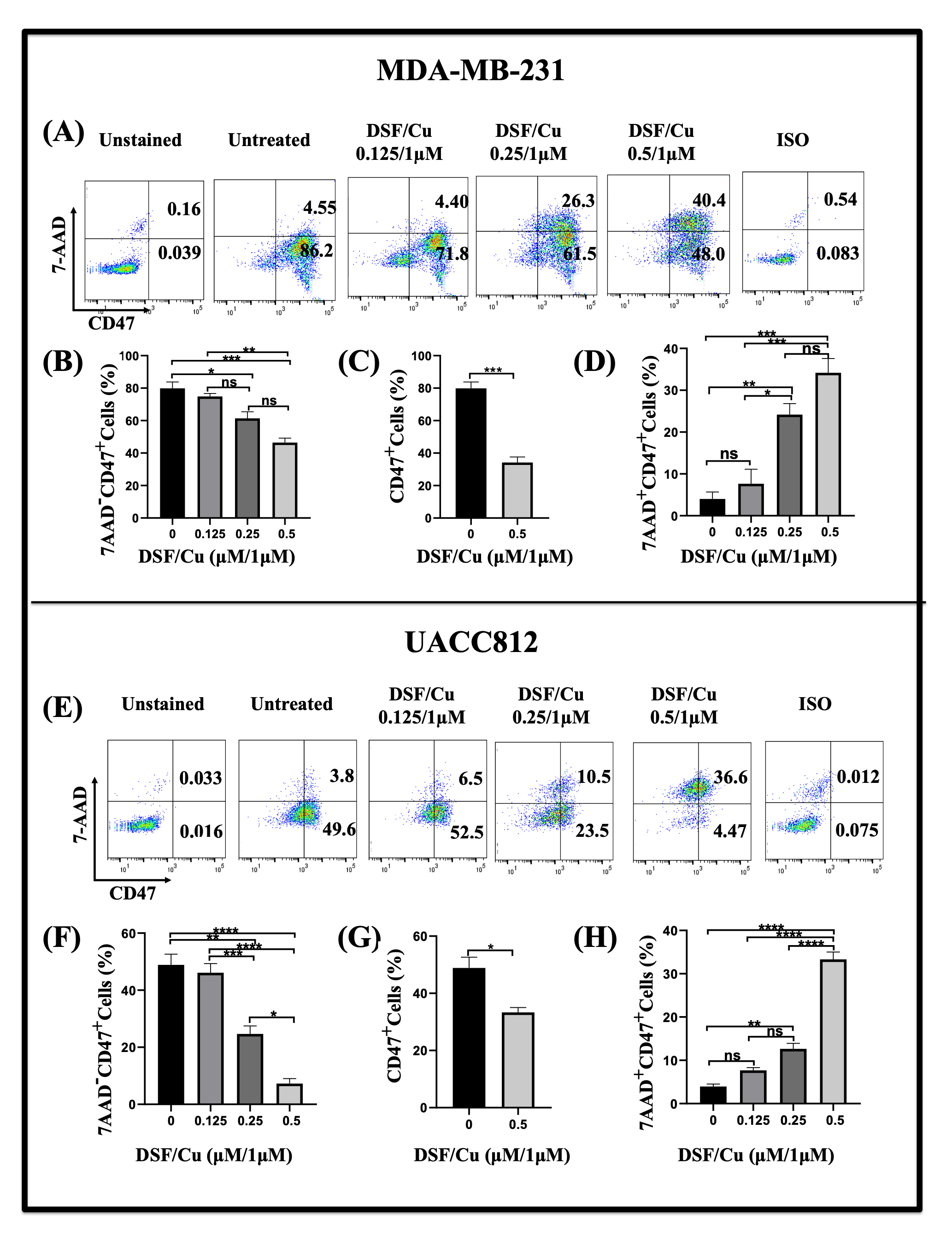

Supplement: Supplementary file 1 — Supplementary Figure 1 [file 41419_2024_6644_MOESM1_ESM.png]

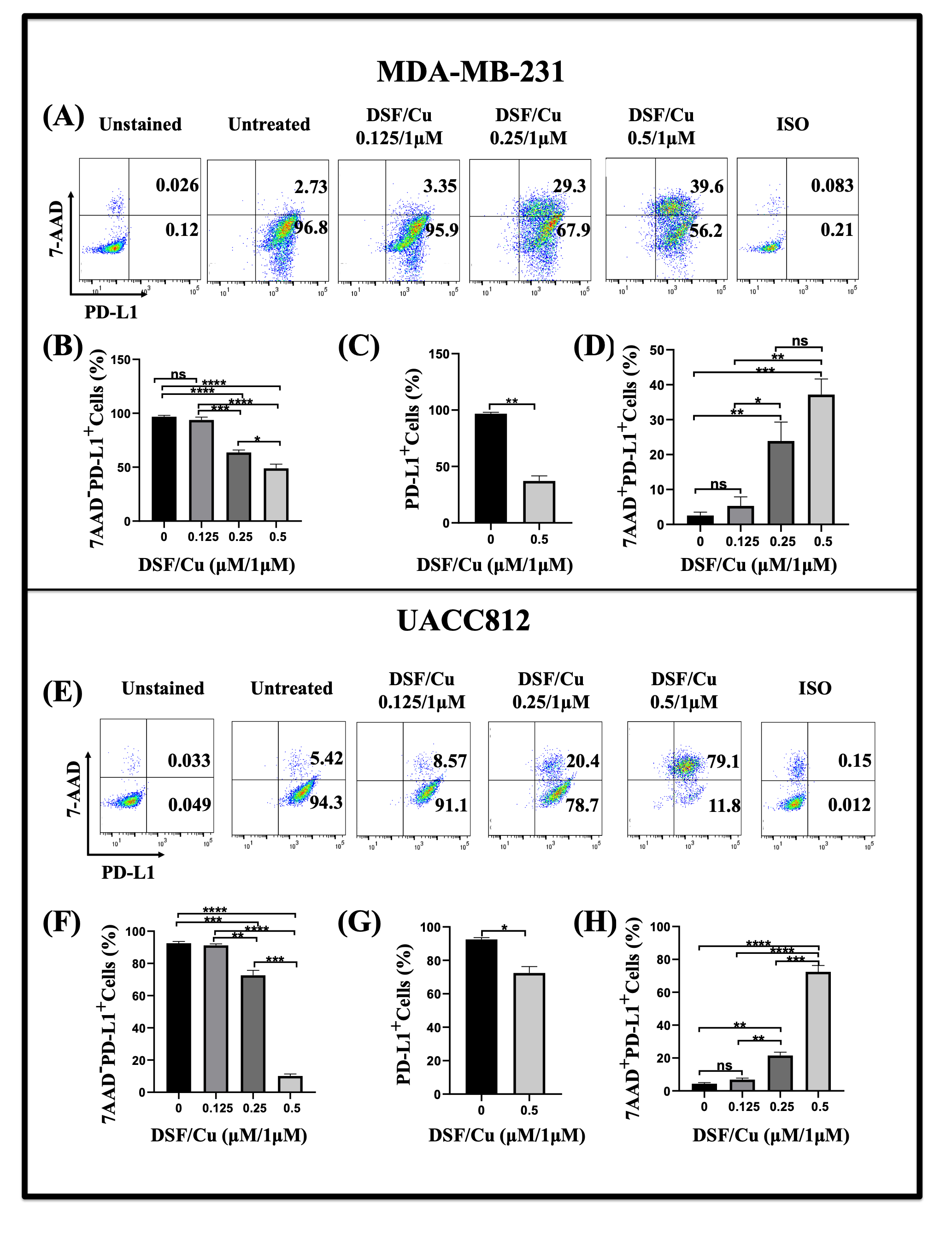

Supplement: Supplementary file 2 — Supplementary Figure 2 [file 41419_2024_6644_MOESM2_ESM.png]

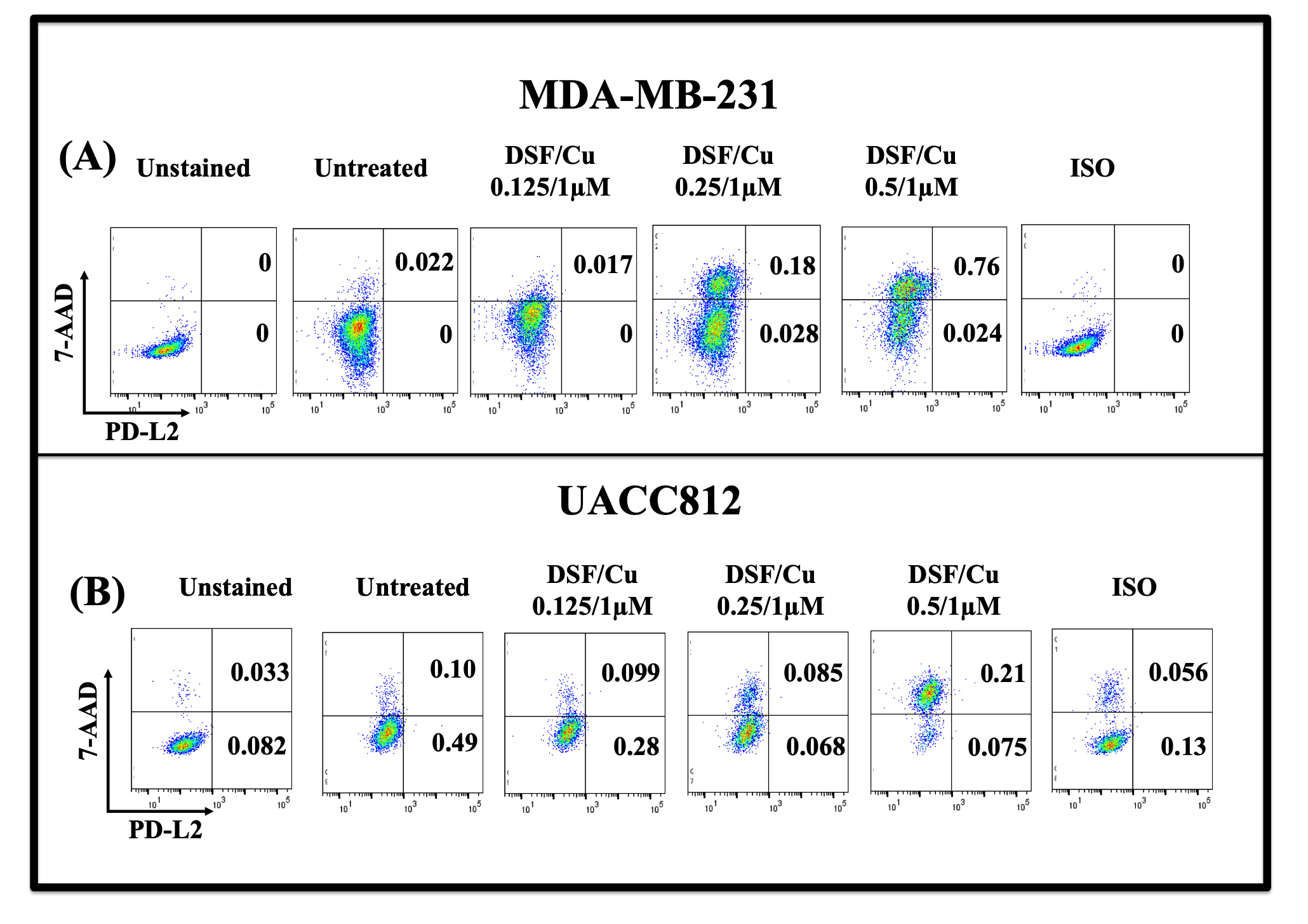

Supplement: Supplementary file 3 — Supplementary Figure 3 [file 41419_2024_6644_MOESM3_ESM.png]
