# Supplementary material for: Turning anecdotal irradiation-induced anticancer immune responses into reproducible in situ cancer vaccines via disulfiram/copper-mediated enhanced immunogenic cell death of breast cancer cells
Source: Cell Death Dis. 2024 Apr 27;15(4):298. doi: 10.1038/s41419-024-06644-3 (PMC11055882; doi:10.1038/s41419-024-06644-3)
Supplement: Supplementary file 5 — Legends for supplementary figures and table [file 41419_2024_6644_MOESM5_ESM.docx]

**Turning anecdotal irradiation-induced anticancer immune responses into reproducible in situ cancer vaccines via disulfiram/copper-mediated enhanced immunogenic cell death of breast cancer cells**

Wei Guo, Lin Jia, Ling Xie, Juliann G. Kiang, Yangyang Wang, Fengfei Sun, Zunwen Lin, Enwen Wang, Yida Zhang, Peigen Huang, Ting Sun, Xiao Zhang, Yingzheng Bian, Tiejun Tang, Jingtian Guo, Soldano Ferrone, and Xinhui Wang

**Supplementary Figures**

**FIGURE LEGENDS**

**sFig. 1. DSF/Cu-induced reduction in CD47 cell surface expression in vitro**. (**A–H**) Cells were treated with DSF at the indicated concentrations and CuCl2 (1 μM) for 24 h in vitro. (**A, E**) human breast cancer MDA-MB-231 (**A**) and UACC812 (**E**) cells were harvested, and aliquots were subjected to flow cytometry analysis for CD47 staining in the presence of 7-AAD, distinguishing viable from dying/dead cells. Representative flow cytometry data are presented (**A, E**). Data of CD47 expression on viable cells (7-AAD^-^CD47^+^) as the mean ± SEM (**B, F**); CD47 expression on untreated viable cells (7-AAD**^-^**CD47^+^) vs. dying/dead cells (7-AAD^+^CD47^+^) induced by the highest DSF/Cu concentration (0.5µM/1 µM ) as the mean ± SEM (**C, G**) and CD47 expression on dying/dead cells (7-AAD^+^CD47^+^) treated by DSF/Cu at the indicated concentrations as the mean ± SEM (**D, H**) are presented. For all flow staining experiments, an isotype matched control antibody was used as a specificity control. All experiments were conducted independently at least 3 times. ****p < 0.0001, ***p < 0.001, **p < 0.01, *p< 0.05, ns: not significant.

**sFig. 2. DSF/Cu-induced reduction in PD-L1 cell surface expression in vitro**. (**A–H**) Cells were treated with DSF at the indicated concentrations and CuCl2 (1 μM) for 24 h in vitro. (**A, E**) human breast cancer MDA-MB-231 (**A**) and UACC812 (**E**) cells were harvested, and aliquots were subjected to flow cytometry analysis for PD-L1 staining in the presence of 7-AAD, distinguishing viable from dying/dead cells. Representative flow cytometry data are presented (**A, E**). Data of PD-L1 expression on viable cells (7-AAD^-^PD-L1^+^) as the mean ± SEM (**B, F**); PD-L1 expression on untreated viable cells (7-AAD**^-^**PD-L1^+^) vs. dying/dead cells (7-AAD^+^PD-L1^+^) induced by the highest DSF/Cu concentration (0.5µM/1 µM ) as the mean ± SEM (**C, G**) and PD-L1 expression on dying/dead cells (7-AAD^+^PD-L1^+^) treated by DSF/Cu at the indicated concentrations as the mean ± SEM (**D, H**) are presented. For all flow staining experiments, an isotype matched control antibody was used as a specificity control. All experiments were conducted independently at least 3 times. ****p < 0.0001, ***p < 0.001, **p < 0.01, *p< 0.05, ns: not significant.

**sFig. 3. Stable negative PD-L2 cell surface expression regardless of DSF/Cu treatment in vitro.** (**A–B**) Cells were treated with DSF at the indicated concentrations and CuCl2 (1 μM) for 24 h in vitro. (**A, B**) human breast cancer MDA-MB-231 (**A**) and UACC812 (**B**) cells were harvested, and aliquots were subjected to flow cytometry analysis for PD-L2 staining in the presence of 7-AAD, distinguishing viable from dying/dead cells. Representative flow cytometry data are presented (**A, B**), illustrating that negative PD-L2 expression did not alter in response to DSF/Cu treatment. For all flow staining experiments, an isotype matched control antibody and an anti-PD-L1 antibody (used for Fig.2.) were used as negative and positive controls. All experiments were conducted independently at least 3 times.

**Supplementary Table 1**

**sTable1. Related to Fig.7F, G.** The original data for cytokines/chemokines in mouse tumors and sera are presented. The Bio-Plex Pro™ Mouse Cytokine Group I Panel 23-Plex was utilized for the assay, which was analyzed using the Luminex H 100™ System.
